# SNRPB-mediated RNA splicing drives tumor cell proliferation and stemness in hepatocellular carcinoma

**DOI:** 10.18632/aging.202164

**Published:** 2020-12-03

**Authors:** Yu-Ting Zhan, Lei Li, Ting-Ting Zeng, Ning-Ning Zhou, Xin-Yuan Guan, Yan Li

**Affiliations:** 1 State Key Laboratory of Oncology in South China, Collaborative Innovation Center for Cancer Medicine, Sun Yat-sen University Cancer Center, Guangzhou 510060, P. R. China; 2 Department of Clinical Oncology, The University of Hong Kong, Hong Kong 852, P. R. China

**Keywords:** SNRPB, hepatocellular carcinoma, RNA splicing, cancer stem cell, glycolysis

## Abstract

Hepatocellular carcinoma (HCC) is one of the leading malignant diseases worldwide, but therapeutic targets for HCC are lacking. Here, we characterized a significant upregulation of Small Nuclear Ribonucleoprotein Polypeptides B and B1 (SNRPB) in HCC via qRT-PCR, western blotting, tissue microarray and public database analyses. Increased SNRPB expression was positively associated with adjacent organ invasion, tumor size, serum AFP level and poor HCC patient survival. Next, we transfected SNRPB into HCC cells to construct SNRPB-overexpressing cell lines, and short hairpin RNA targeting SNRPB was used to silence SNRPB in HCC cells. Functional studies showed that SNRPB overexpression could promote HCC cell malignant proliferation and stemness maintenance. Inversely, SNRPB knockdown in HCC cells caused inverse effects. Importantly, analysis of alternative splicing by RNA sequencing revealed that SNRPB promoted the formation of AKT3-204 and LDHA-220 splice variants, which activated the Akt pathway and aerobic glycolysis in HCC cells. In conclusion, SNRPB could serve as a prognostic predictor for patients with HCC, and it promotes HCC progression by inducing metabolic reprogramming.

## INTRODUCTION

Primary liver cancer is one of the most common cancers with a poor prognosis. Currently, the 5-year survival rate of primary liver cancer patients is just 18% worldwide [1, 2]. Hepatocellular carcinoma (HCC), the most common pathological type of liver cancer, accounts for approximately 80% of cases [3]. Hepatocarcinogenesis is a multistep, multifactor process that includes the activation of oncogenes and the suppression of tumor suppressor genes [4]. Therefore, the identification of new driver genes is necessary to increase the effects of HCC treatments and to improve the outcomes of HCC patients.

The spliceosome is an organelle-like complex and is mainly distributed in the nucleus. The standard spliceosome is usually composed of five small nuclear ribonucleoproteins (snRNPs), namely, U1, U2, U4, U5, and U6, and more than 150 spliceosome-associated proteins (SAPs). These snRNPs and SAPs participate in the splicing of precursor messenger RNA (premRNA), including removing introns from premRNA by excision reactions and splicing together exons according to a certain rule. Eukaryotic genes are usually broken genes, and the exon sequence encoding the protein is separated by nonencoding sequences; thus, the premRNAs produced by transcription cannot be directly translated into proteins [5, 6]. Previously, it was thought that the premRNAs of most genes were spliced in a fixed manner to produce mature mRNA molecules that were then translated into proteins. However, many genes have different splicing sites and patterns in their premRNAs, resulting in the generation of different mRNA splice isomers. This process is called alternative splicing [7]. Alternative splicing of RNA is a more flexible strategy for the posttranscriptional regulation of genes, which greatly increases the diversity of proteins [7–9].

Small Nuclear Ribonucleoprotein Polypeptides B and B1 (SNRPB or smB/B′), a core component of the spliceosome, is involved in regulating alternative splicing of premRNAs. There are three known transcripts of the *SNRPB* gene: splice variants 1 (V1) and 2 (V2) encode the smB' and smB proteins, respectively, and splice variant 3 (V3) undergoes nonsense degradation. The amino acid sequences of the two proteins (smB'/smB) encoded by the *SNRPB* gene are very similar, and they form a part of the core component of the spliceosome [10]. In fact, the smB 'and smB proteins are members of a group of proteins with similar RNA-binding proteins that contain Sm sites [11]. The *SNRPB* gene was found to be related to brain-cochlear-mandibular syndrome, systemic lupus erythematosus and Crohn's disease [12–15]. For instance, deficiency of *SNRPB* expression during embryonic and juvenile stages can cause the malformation observed in brain-cochlear-mandibular syndrome [13]. The Sm protein expressed by somatic cells can cause an autoimmune response in the occurrence of systemic lupus erythematosus [16, 17]. Recently, it has been reported that other Sm proteins, such as SNRPD3 and SNRPE, were upregulated in nonsmall cell lung cancer, promoting cancer development [18, 19]. SNRPB may also be a potential oncogene for nonsmall cell lung cancer and glioblastoma [18–20]. By analyzing The Cancer Genome Atlas (TCGA) database, we found that SNRPB was significantly upregulated in HCC, and dysregulation of SNRPB was associated with worse survival of HCC patients. However, the role of SNRPB in HCC progression needs to be explored.

In this study, we found that the mRNA and protein levels of SNRPB were upregulated in HCC tissues compared with adjacent normal liver tissues and that SNRPB was a potential marker of poor prognosis in HCC patients. We characterized the functions of SNRPB in HCC by both *in vitro* and *in vivo* studies and showed that it contributed to HCC cell proliferation and stemness. Furthermore, RNA sequencing analysis of alternative splicing revealed that SNRPB activated the Akt pathway and aerobic glycolysis in HCC cells by increasing the formation of the *AKT3-204* and *LDHA-220* splice variants. Therefore, SNRPB plays a crucial role in HCC progression.

## RESULTS

### Aberrantly high expression of SNRPB in HCC

**Figure 1:**
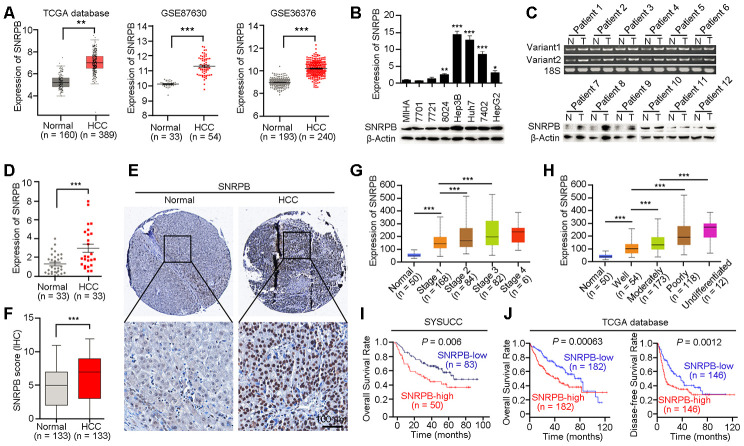
**Overexpression of SNRPB predicts poor survival of HCC patients.** (**A**) The expression levels of *SNRPB* in normal liver tissues and HCC tissues were analyzed based on TCGA database and GEO datasets (GSE87630 and GSE36376). (**B**) Real-time quantitative PCR (qRT-PCR, upper panel) and western blotting (lower panel) were used to examine the expression of SNRPB in HCC cell lines and immortalized hepatocytes (MIHA). 18S or β-Actin served as the loading controls. (**C**) The expression levels of SNRPB in paired HCC tumor (T) and normal liver (N) tissues were examined by RT-PCR (upper panel) and western blotting (lower panel). Two splicing variants of SNRPB were analyzed by RT-PCR, and 18S served as the loading control. (**D**) qRT-PCR was used to examine the expression levels of *SNRPB* in HCC tissues and the corresponding normal liver tissues (n = 33). (**E**) Representative images of IHC staining of SNRPB in paired normal liver and HCC tissues. (**F**) IHC staining scores of SNRPB in HCC tissues and the corresponding normal liver tissues (n = 133). (**G**, **H**) SNRPB expression is related to HCC cancer stages (**G**) and differentiation grades (**H**) in TCGA database. (**I**, **J**) Kaplan–Meier survival curves showed that SNRPB expression level was negatively correlated with HCC prognosis, as analyzed by HCC tissue microarray (**I**) and TCGA database (**J**). In all panels, **P < 0.01, ***P < 0.001.

SNRPB is a key subunit of the spliceosome that is involved in regulating the alternative splicing of the premRNA, but its role in cancer progression is unclear [21]. Based on TCGA database analysis, we found that the mRNA expression level of *SNRPB* was significantly higher in HCC tissues than in adjacent normal liver tissues (Figure 1A, left panel). Considering that the samples in TCGA database come from the United States, and may be inconsistent with samples from China, we confirmed the higher expression of *SNRPB* in HCC tissues compared to normal liver tissues in two Chinese-derived GEO datasets (GSE87630 and GSE36376, Figure 1A, middle and right panels). Next, quantitative reverse transcription PCR (qRT-PCR) and western blotting were used to detect the expression of SNRPB in an immortalized hepatic epithelial cell line (MIHA) and seven HCC cell lines. The results showed that the expression level of SNRPB was higher in the cell lines BEL7402, Hep3B, 8024, huh7 and HepG2 than in the MIHA cell line (Figure 1B). A previous study reported that the *SNRPB* gene had two variants (*SNRPB-V1* and *SNRPB-V2*) in somatic cells with similar expression trends in humans [22]. In HCC, qRT-PCR indicated that both *SNRPB-V1* and *SNRPB-V2* were more highly expressed in tumor tissues than in adjacent normal liver tissues (Figure 1C, upper panel). The increased level of the SNRPB protein in human HCC tissues was also confirmed by western blotting analysis (Figure 1C, lower panel) and qRT-PCR (Figure 1D).

### High level of SNRPB correlates with unfavorable prognosis of HCC patients

A HCC tissue microarray, including 133 paired adjacent normal liver tissues and HCC tissues, was used to evaluate the correlation of SNRPB expression with clinicopathological characteristics. The IHC staining results showed an increased level of SNRPB in HCC tissues compared to normal liver tissues (Figure 1E, 1F). Additionally, high expression of SNRPB was positively correlated with tumor size (*P* = 0.001), adjacent organ invasion (*P* = 0.003) and serum AFP level (*P* = 0.004, Table 1). Moreover, the gene *SNRPB* expression levels were gradually increased from the early stage to stage III of HCC as well as in the well-differentiated group and the undifferentiated group based on TCGA dataset (Figure 1G, 1H). To further analyze the correlation of clinical features or SNRPB levels with the overall survival of HCC patients, we performed a univariate Cox regression analysis and found that higher SNRPB expression (*P* < 0.001), peripheral organ infiltration (*P* < 0.001), high AFP level (*P* < 0.001) and tumor embolus (*P* < 0.001) were associated with the poor HCC patient prognosis (Supplementary Figure 1A). Multivariate Cox regression analysis showed that high AFP level and tumor embolus were independent prognostic factors for patients with HCC (Supplementary Figure 1B). Moreover, Kaplan-Meier analysis revealed that upregulation of SNRPB indicated the poor survival of HCC patients (*P* = 0.006, Figure 1I). Moreover, survival analysis using TCGA clinical data showed undesirable overall survival (*P* = 0.00063) and progression-free survival (*P* = 0.0012) in HCC patients with high SNRPB expression compared with HCC patients with low SNRPB expression (Figure 1J). Therefore, SNRPB plays an aggressive role during HCC malignant progression.

**Table 1:** Clinicopathological correlation of SNRPB expression in HCC.

**Clinicopathological Features**	**Cases**	**SNRPB expression**		***P* value**
		**Low**	**High**	
Gender				
Male	126	79(62.7)	47(37.3)	1
Female	7	4 (57.1)	3 (42.9)	
Age(years)				
≤60	105	63(60.0)	42(40.0)	0.267
>60	28	20(70.4)	8 (28.6)	
Hepatitis B surface Ag				
Negative	17	14(82.4)	3 (17.6)	0.065
Positive	115	68(59.1)	47(40.9)	
Serum AFP (ng/ml)				
<400	79	57(72.2)	22(27.8)	**0.004**
≥400	53	25(47.2)	28(52.8)	
Tumor size (cm)				
<5	36	31(44.4)	5 (55.6)	**0.001**
≥5	95	51(53.7)	44(46.3)	
Cirrhosis				
Absent	22	14(63.6)	8 (36.4)	0.872
Present	110	68(61.8)	42(38.2)	
Tumor encapsulation				
Absent	50	29(58.0)	21(42.0)	0.446
Present	82	53(64.6)	29(35.4)	
Microsatellite formation				
Absent	103	64(62.1)	39(37.9)	0.995
Present	29	18(62.1)	11(37.9)	
Adjacent organ invasion				
Absent	105	72(68.6)	33(31.4)	**0.003**
Present	27	10(37.0)	17(63.0)	
Thrombus				
Absent	112	73(65.2)	39(34.8)	0.074
Present	19	8 (42.1)	11(57.9)	
Recurrence				
No	117	75(64.1)	42(35.9)	0.285
Yes	16	8 (50.0)	8 (50.0)	

Statistical significance (P < 0.05) is shown in bold.

### SNRPB overexpression promotes HCC cell proliferation

**Figure 2:**
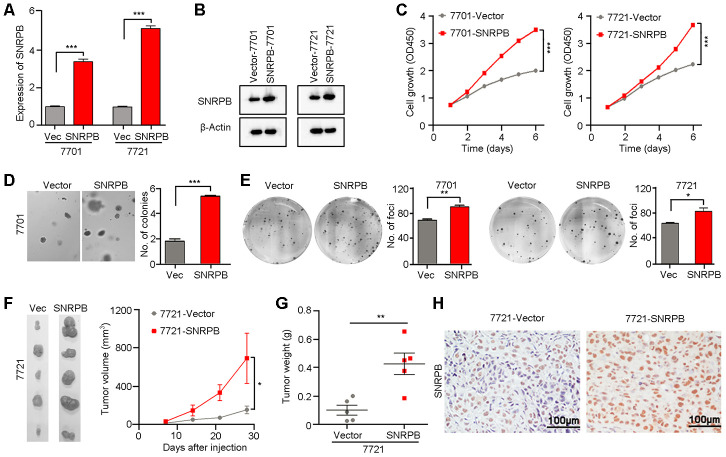
**Overexpression of SNRPB promotes tumor growth in HCC cells.** (**A**, **B**) qRT-PCR (**A**) and western blotting (**B**) were used to confirm the overexpression of SNRPB in *SNRPB*-transfected 7701 and 7721 cells. 18S or β-Actin served as the loading control. (**C**) Cell growth rates of empty vector- or *SNRPB*-transfected 7701 or 7721 cells. (**D**, **E**) Representative images of increased colony formation in soft agar (**D**) and foci formation in monolayer culture (**E**) induced by *SNRPB* overexpression in 7701 or 7721 cells. The number of colonies or foci in the vector-transfected and *SNRPB-*overexpressing groups are summarized in the right panel. (**F**) Images and growth curves of the xenograft tumors formed in nude mice injected with *SNRPB*- and empty vector-transfected 7721 cells (*n* = 5). (**G**) The weights of xenograft tumors derived from *SNRPB*- and empty vector-transfected 7721 cells are summarized. (**H**) IHC staining was used to confirm the level of SNRPB in xenograft tumors. Scale bars = 100 μm. In all panels, *P < 0.05, **P < 0.01, ***P < 0.001.

Given that a high level of SNRPB is correlated with larger tumor volume in HCC, we hypothesize that SNRPB promotes tumor cell malignant proliferation during HCC progression. Moreover, the level of *SNRPB* was also positively related to the levels of the proliferative markers *Ki-67* and *PCNA* in HCC based on TCGA database (Supplementary Figure 2). Hence, to confirm the hypothesis, the HCC cell lines 7701 and 7721 with relatively lower levels of SNRPB expression were transfected with *SNRPB* or empty vector via lentivirus. The *SNRPB* expression levels were then evaluated by qRT-PCR (Figure 2A) and western blot (Figure 2B). Functional studies showed that the cell growth rate in the *SNRPB*-transfected cells was higher than that in the control cells (*P* < 0.001, Figure 2C). Colony formation in soft agar and foci formation in 2D culture plate assays showed that the formation frequency of microspheres or foci were significantly increased in *SNRPB*-overexpressing cells compared with vector-transfected cells (Figure 2D, 2E). A xenograft tumor assay in nude mice indicated that the volumes and weights of transplanted tumors derived from *SNRPB*-transfected 7721 cells were larger than tumors developed from vector cells (Figure 2F–2G). Moreover, the expression levels of SNRPB in xenograft tumors were confirmed by IHC staining (Figure 2H). Therefore, overexpression of SNRPB promotes HCC malignant progression by facilitating tumor cell growth.

### Silencing SNRPB reduces tumor growth

**Figure 3:**
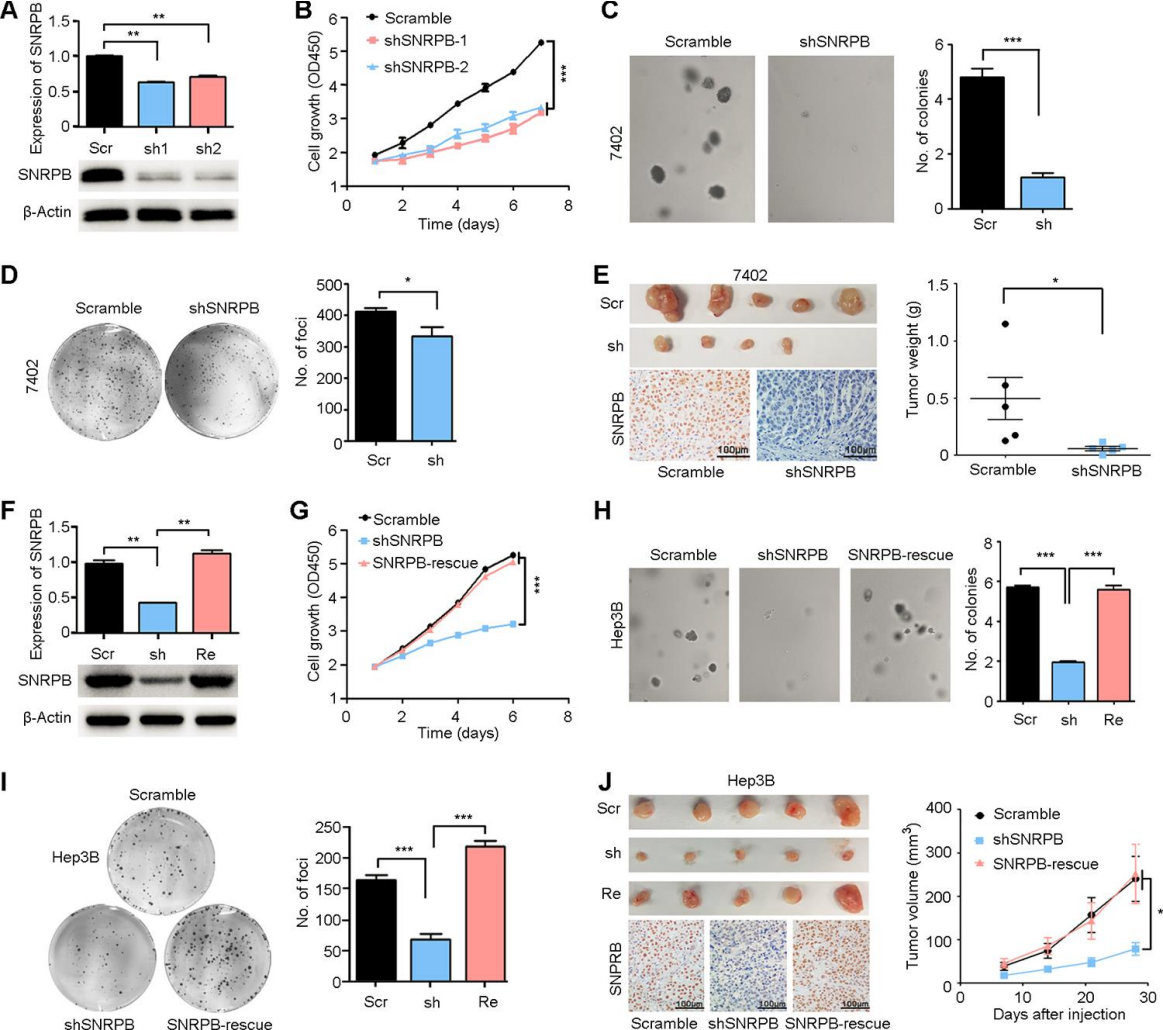
**Silencing SNRPB inhibits HCC cell growth *in vitro* and *in vivo*.** (**A**) qRT-PCR and western blotting analyses indicating the silencing of *SNRPB* with shRNAs (shSNRPBs) in 7402 cells. 18S or β-Actin served as the loading control. (**B**) XTT assay showing that the cell growth rate of 7402 cells was inhibited by shSNRPB. (**C**) Representative images of decreased colony formation induced by shSNRPB in soft agar assays. The results are summarized in the right panel. (**D**) Representative image of foci formation in monolayer culture of 7402 cells with silenced *SNRPB*. The numbers of foci are summarized in the right panel. (**E**) Images of the xenograft tumors formed in nude mice injected with shSNRPB- and scramble-transfected cells. The weights of xenograft tumors are summarized in the right panel. IHC staining was performed to confirm the expression of SNRPB in xenograft tumors (lower panel). Scale bars = 100 μm. (**F**) qRT-PCR and western blotting analyses showing the expression levels of SNRPB in Hep3B cells transfected with *SNRPB*-shRNA (shSNRPB) and *SNRPB*-overexpressing vector for rescue (*SNRPB*-rescue). 18S or β-Actin served as the loading control. (**G**–**I**) Cell growth curves (**G**), colony formation (**H**) and foci formation (**I**) assays showed that transfection with *SNRPB* could rescue the cell growth inhibited by shSNRPB in Hep3B cells. (**J**) Images of the xenograft tumor formed in nude mice injected with scramble vector-, shSNRPB- and *SNRPB*-rescue-transfected cells. The volume curves of xenograft tumors are summarized in the right panel. IHC staining was performed to confirm the expression of SNRPB in xenograft tumors (lower panel). Scale bars = 100 μm.

First, two high-efficiency targeted shRNAs (sh*SNRPB*-1 and sh*SNRPB*-2) were stably transfected into the HCC cell line 7402, which expressed SNRPB at high levels. qRT-PCR and western blot assays were performed to confirm the knockdown efficiency of shRNAs in 7402 cells (Figure 3A). Functional assays revealed that *SNRPB* silencing inhibited the proliferation activity of 7402 cells (*P* < 0.001, Figure 3B) and the frequencies of colony formation *in vitro* (Figure 3C, 3D). In addition, an *in vivo* xenograft tumor assay suggested that knockdown of *SNRPB* impaired its tumorigenicity (Figure 3E, upper panel). Knockdown of *SNRPB* was also confirmed by IHC staining in xenograft tumors derived from sh*SNRPB*-transfected cells compared with tumors induced by scramble control cells (Figure 3E, lower panel).

In addition, rescue of *SNRPB* was performed in sh*SNRPB*-transfected Hep3B cells to further confirm the pro-proliferation role of *SNRPB* (Figure 3F). A cell growth assay showed that transfection with *SNRPB* could rescue cell growth in *SNRPB*-silenced Hep3B cells (Figure 3G). Sphere formation in soft agar (Figure 3H), foci formation in monolayer culture (Figure 3I) and tumorigenesis in nude mice (Figure 3J, upper panel) indicated that knockdown of *SNRPB* could suppress tumor cell growth, and *SNRPB* transfection rescued the proliferation of Hep3B cells. Moreover, the recuperative expression of SNRPB in xenograft tumors derived from *SNRPB*-rescued cells was confirmed by IHC staining (Figure 3J, lower panel). In conclusion, these results indicate the key oncogenic role of SNRPB in HCC.

### SNRPB is involved in maintaining cell stemness in HCC

**Figure 4:**
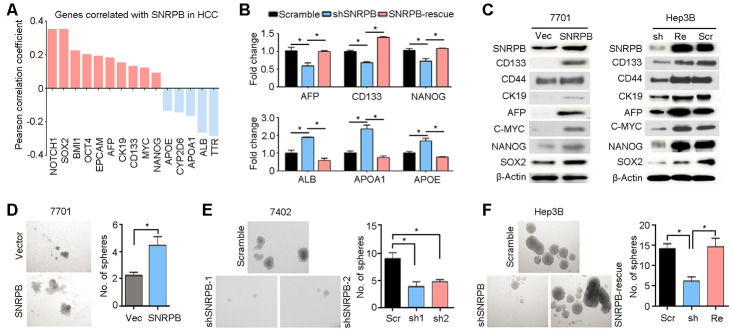
**SNRPB maintains HCC cell stemness.** (**A**) Expression correlation analysis indicating the genes correlated with *SNRPB* in HCC based on TCGA database. (**B**) The expression levels of genes associated with HCC stemness (*AFP*, *CD133* and *NANOG*) and markers of differentiated hepatocytes (*ALB*, *APOA1* and *APOE*) in Hep3B cells transfected with scramble vector, shSNRPB or *SNRPB*-rescue were tested by qRT-PCR. (**C**) Western blotting analysis indicates the expression levels of proteins associated with HCC stemness in 7701 cells transfected with vector and SNRPB (left panel) and Hep3B cells transfected with scramble vector, shSNRPB or *SNRPB*-rescue (right panel). β-Actin served as the loading control. (**D**–**F**) Stem cell sphere formation assays were performed on 7701 cells transfected with vector and *SNRPB* (**D**), 7402 cells transfected with scramble and shSNRPB (E) and Hep3B cells with scramble vector, shSNRPB or *SNRPB*-rescue (**F**). In all panels, *P < 0.05.

Clinicopathological data correlation analysis has suggested that the SNRPB level was significantly positively associated with the serum AFP level (Table 1). AFP protein was associated with cell stemness in HCC [23, 24]. Hence, we analyzed TCGA database and showed that *SNRPB* expression levels were positively correlated with most well-recognized markers of cell stemness (such as *CD133*, *EPCAM*, *CK19* and *AFP*) and negatively correlated with markers of hepatocyte epithelial cells (including *ALB* and *TTR*) in HCC (Figure 4A). Moreover, qRT-PCR analyses showed that knockdown of *SNRPB* in Hep3B cells could reduce the expression levels of stemness-associated genes (*AFP*, *CD133* and *NANOG*) and induce the expression of differentiated hepatocyte markers (*ALB*, *APOA1* and *APOE*) (Figure 4B). Western blotting also indicated that protein expression levels of HCC stem markers (CD133, CD44, CK19 and AFP) and stemness-associated transcription factors (c-Myc, Nanog and Sox2) in 7701 cells were downregulated after overexpression of *SNRPB* (Figure 4C). Furthermore, rescued *SNRPB* expression in Hep3B cells significantly increased the levels of these proteins in HCC cells (Figure 4C). Therefore, a stem cell sphere formation assay was performed on 7701 cells transfected with vector or *SNRPB*, 7402 cells transfected with scramble and shSNRPBs, and Hep3B cells transfected with scramble vector, shSNRPB or *SNRPB*-rescue (Figure 4D–4F). The results showed that overexpression of *SNRPB* enhanced the frequency of sphere formation in 7701 cells (Figure 4D). Inversely, silencing *SNRPB* in 7402 and Hep3B cells decreased the number of stem spheres, and rescue of *SNRPB* in Hep3B cells significantly recovered the sphere formation ability (Figure 4E, 4F). In addition, hematoxylin-eosin staining indicated a more disarranged and irregular tissue organization in xenografts from SNRPB-overexpressing 7701 cells than that from wild-type cells (Supplementary Figure 3A). Intracellular lipid droplet accumulation has been reported to be a hallmark of HCC stem cells [25]. Our Oil Red O staining also showed increased lipid droplet storage in 7701 cells after overexpression of *SNRPB* (Supplementary Figure 3B). The number of lipid droplets in Hep3B cells was reduced when *SNRPB* was silenced (Supplementary Figure 3C). Taken together, these research data suggest that SNRPB promotes HCC progression by regulating cell stemness.

### SNRPB activates the AKT pathway and glycolysis via alternative splicing regulation

**Figure 5:**
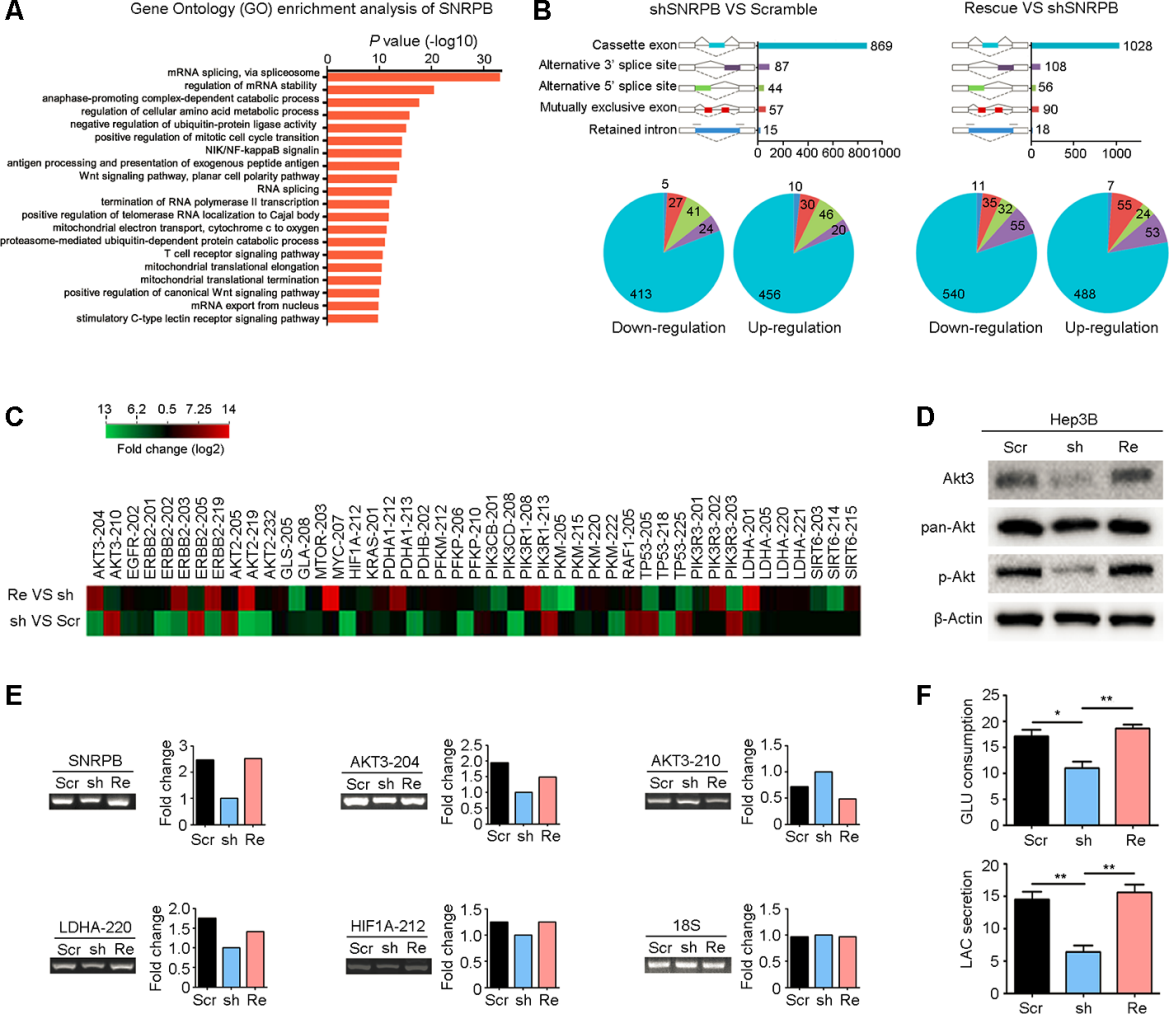
**SNRPB activates the Akt pathway and glycolysis via alternative splicing regulation.** (**A**) Gene Ontology (GO) enrichment analysis (biological process) of *SNRPB* using the Coexpedia internet tool (http://www.coexpedia.org/) based on public GEO datasets. (**B**) Module plot indicates five major types of alternative splicing in Hep3B cells transfected with scramble, shSNRPB and *SNRPB*-rescue (upper panel). Pie chart shows the upregulation or downregulation of five alternative splicing events (lower panel). (**C**) The heatmap shows the genes associated with carbon metabolism with alternative splicing in Hep3B cells after *SNRPB* knockdown or rescue. (**D**) The levels of Akt3, pan-Akt and p-Akt in Hep3B cells transfected with scramble, shSNRPB and *SNRPB*-rescue were detected by western blotting, and β-actin served as the loading control. (**E**) Some specific altered transcripts were confirmed by RT-PCR in Hep3B cells transfected with scramble, shSNRPB and *SNRPB*-rescue. The gene expression bands were quantified with ImageJ software (https://imagej.net/). (**F**) Glucose consumption and lactate secretion were decreased by *SNRPB* silencing but could be recovered by rescue of *SNRPB*. *P < 0.05, **P < 0.01.

To explore the mechanism by which SNRPB regulates HCC progression, we performed Gene Ontology Enrichment analysis (biological process) with Coexpedia [26]. Analysis results showed that SNRPB was significantly related to mRNA splicing (Figure 5A). Next, RNA sequencing was used to explore the alternative splicing events regulated by SNRPB; these splicing events include five recognized forms of RNA splicing, including exon cassette, alternative 3’ or 5’ splices, mutually exclusive exon and retained intron, that generate different gene transcripts in humans [27]. Analysis showed that knockdown of *SNRPB* in Hep3B cells induced the upregulation of 562 transcripts and the downregulation of 510 transcripts. Rescue of *SNRPB* upregulated 673 variants and downregulated 627 variants (Figure 5B). Therefore, SNRPB played a key role in variant formation via alternative splicing regulation in HCC.

Next, we analyzed the splicing events and variants regulated by SNRPB and found that these transcripts with significant differences were associated with the tumor carbon metabolism pathway. According to the KEGG database, 46 transcripts of 22 metabolism-related genes were significantly changed after knockdown of *SNRPB* or rescue of *SNRPB* (Figure 5C). First, western blotting confirmed that silencing *SNRPB* in Hep3B cells significantly reduced the expression of Akt3 translated from the splice variant *AKT3-204* (Figure 5D). Moreover, the levels of phosphorylated and total Akt were also decreased after *SNRPB* knockdown, which could be recovered by rescued of *SNRPB* in Hep3B cells (Figure 5D). RT-PCR also confirmed the expression levels of *AKT3-204*, *AKT3-210* and metabolism-related variants *LDHA-220* and *HIF1A-212* regulated by SNRPB in Hep3B cells (Figure 5E). In addition, LDHA is the rate-limiting step in catalyzing glycolysis [28]. We silenced and then rescued *SNRPB* to observe the effects on glucose consumption and lactic acid secretion of Hep3B cells. The results showed that glucose consumption and lactate secretion of Hep3B cells were significantly decreased after *SNRPB* knockdown and were increased after rescue of *SNRPB* (Figure 5F). In addition, the ATP synthesis efficiency of *SNRPB*-silenced Hep3B cells was also reduced compared to control cells (Supplementary Figure 4). Collectively, these data support the idea that SNRPB enhances glycolysis in HCC cells.

## DISCUSSION

SNRPB, as a component of the spliceosome, is implicated in alternative splicing. Recently, it has been reported that dysregulation of SNRPB is involved in human cancers, such as nonsmall cell lung cancer [20], glioblastoma [29] and cervical cancer [30]. These studies proved that overexpression of SNRPB was correlated with poor prognosis of cancer patients. In this study, we also showed that high expression of SNRPB predicted poor survival of patients with HCC. Therefore, SNRPB-mediated alternative splicing promotes cancer progression and may be used as a prognostic marker in HCC. However, the mechanism of SNRPB upregulation in HCC cells is unclear. One recent work demonstrated that c-Myc directly drove the transcription of *SNRPB* in HCC, as shown by luciferase reporter and chromatin immunoprecipitation assays [31]. In addition, there are two splice variants of the human *SNRPB* gene (*SNRPB-V1* and S*NRPB-V2*), but their expression profiles in cancer tissues have not been explored [22]. Using specific primers, we analyzed the expression levels of *SNRPB-V1* and S*NRPB-V2* in HCC tissues. The RT-PCR results showed no significant differences between the levels of these two variants, and both were highly upregulated in HCC tissues compared with normal liver tissues. These findings strongly suggest a potential aggressive role of SNRPB in HCC progression.

Emerging evidence has suggested that *SNRPB* overexpression promoted cancer cell proliferation and metastasis [20, 30, 31]. Similar to previous studies, the present study showed that *SNRPB* silencing decreased proliferative activity and cell stemness. In addition, we found that *SNRPB* was significantly positively correlated with cell stemness markers, such as *CD133*, *AFP* and *EpCAM*, based on TCGA database. SNRPB is transcriptionally upregulated by c-Myc in HCC cells [31]. Importantly, c-Myc has biological functions in stemness maintenance, and amplification or overexpression of the *MYC* gene leads to hepatocarcinogenesis [32, 33]. Hence, SNRPB may be involved in c-Myc-induced hepatocarcinogenesis. Our study showed that overexpression of SNRPB upregulated the expression of c-Myc at the protein level. Therefore, SNRPB and c-Myc form a positive feedback loop of expression regulation, which promotes HCC progression.

SNRPB is one of the components of the spliceosome that affects the splicing activity of the premRNA and the expression of tumor-related genes. Using online bioinformatics tools to perform GO analysis of *SNRPB* coexpressed genes in the GEO database, we confirmed that *SNRPB* was closely correlated with RNA splicing. In eukaryotes, genes often consist of noncoding introns and protein-coding exons. PremRNA splicing is an essential process for mRNA maturation, during which introns are removed from premRNA, and exons are spliced together to form mature mRNA [34]. Alternative splicing by selecting different combinations of protein-coding exons from a premRNA produces variably spliced mature mRNAs, which greatly increases the diversity of genes at the posttranscriptional level [7, 35, 36]. Recently, the alternative splicing of premRNA in tumorigenesis has been given more attention due to the improvements of RNA sequencing. In this study, we revealed the decreased frequency of RNA splicing events after knockdown of *SNRPB* in HCC cells.

The alternative splicing of premRNAs regulated by *SNRPB* is involved in carbon metabolism-related pathways. Carbon metabolism is one of the foundations of cellular metabolism, which includes sugar metabolism, fatty acid metabolism, nucleotide metabolism and other metabolic methods [37]. Subsequent experiments showed that the levels of total Akt, Akt3 and phosphorylated Akt were downregulated after *SNRPB* silencing, which indicated that *SNRPB* enhanced Akt pathway activity. The increased activity of Akt signaling promotes tumor growth [38]. In particular, *AKT3-204*, *HIF1A-212* and *LDHA-220* were downregulated after knockdown of *SNRPB*, while *AKT3-210* was upregulated in HCC cells. It is worth noting that the results of *AKT3-204* and *AKT3-210* are completely opposite. *AKT3-204* encodes the Akt3 protein, and *AKT3-210* undergoes nonsense degradation. Therefore, these data suggest that SNRPB activates the key carbon metabolism pathways during HCC progression. In addition, *HIF1A-212* encodes the HIF-1α protein, a transcription factor that promotes the expression of genes such as *MYC* and *ERK* [39]. *LDHA-220* translates into lactate dehydrogenase, LDHA, and the activity of LDHA can be used as one of the indicators of glycolysis activity [40]. Our findings showed that SNRPB increased glucose consumption and lactic acid production, suggesting that SNRPB promotes glycolysis in HCC cells.

SNRPB, as a core component of the spliceosome, plays an oncogenic role in cancers. One research group recently determined that Ras-related protein Rab-26 (RAB26) was involved in SNRPB-mediated cell growth and metastasis in lung cancer. These authors demonstrated that SNRPB regulated the alternative splicing of *RAB26* premRNA [20]. Moreover, SNRPB could also directly interact with p53 protein, which promoted cervical cancer cell survival, migration and invasion [30]. Here, we confirmed that upregulation of SNRPB contributes to HCC cell proliferation and stemness maintenance by activating carbon metabolism-associated genes, such as *AKT3-204*, *HIF1A-212* and *LDHA-220*. However, a greater understanding of SNRPB-mediated alternative splicing is needed to improve the treatment of HCC patients.

## MATERIALS AND METHODS

### Plasmid construction, lentivirus packaging and cell transfection

The coding sequence of human *SNRPB* was cloned into the lentiviral expression vector pReceiver-LV105 (GeneCopoeia, Rockville, MD) for the exogenous overexpression of *SNRPB*. Short hairpin RNAs (shRNAs, sh1: 5'-GGCCTATGAAACTGGTTTATA-3'; sh2: 5'-GCCAAAGAACTCCAAACAAGC-3') targeting *SNRPB* were constructed into the lentiviral interference vector psi-LVRU6GP (GeneCopoeia, Rockville, MD). Empty vectors were also transfected as negative controls. Lentivirus packaging was performed with the Lenti-Pac™ HIV Expression Packaging Kit (#LT002, GeneCopoeia, Rockville, MD) according to the manufacturer’s instructions. Viral supernatant was harvested for the transfection of HCC cells 72 hours after packaging. Polybrene (10 μg/ml, #H9268, Sigma-Aldrich, Burlington, MA) was added to the culture medium to improve transfection efficiency. For the rescue experiments, a LV105 vector containing *SNRPB* was transfected into *SNRPB*-silenced Hep3B cells. The cell clones with stable overexpression or silencing of *SNRPB* were selected with puromycin treatment (2 μg/ml, #P8833, Sigma, Burlington, MA) 72 hours after transfection. The expression level of *SNRPB* was analyzed by qRT-PCR and western blotting.

### Immunohistochemical (IHC) staining

IHC staining was performed according to a standard procedure [41]. In brief, tissue sections were deparaffinized by pure xylene, rehydrated with graded ethanol (100%, 95%, 75% and 50%) and then rinsed with distilled water. Hydrogen peroxide (3% in distilled water) was used to block the endogenous peroxidase activity of the tissues at room temperature for 15 min. Next, tissue slides were high-pressure treated and boiled in a 10 mM citrate buffer (pH=6.0) for 15 min for antigen retrieval. Nonspecific antibody binding was blocked with 5% bovine serum albumin at room temperature for 30 min. The slides were incubated with monoclonal rabbit anti-human SNRPB (#sc-271094, Santa Cruz Biotechnology, 1:200 dilution) overnight at 4° C in a humidified chamber. Tissue sides were washed thrice with PBS and were incubated with horseradish peroxidase (HRP)-conjugated secondary antibody at 37° C for 30 min. Tissue slides were washed thrice with PBS again and were stained with DAB substrate (Dako). Representative images of IHC staining were captured with a light microscope (Olympus, Lake Success, NY).

### Western blotting

Fresh tissue homogenates or tumor cell lysates were lysed in ice-cold RIPA (Cell Signaling Technology) supplemented with 1 mM phenylmethylsulfonyl fluoride (Roche) and 1% protease inhibitor cocktail (Roche). Protein samples (20 μg per sample) were separated on SDS-PAGE gels and then transferred onto polyvinylidene difluoride membranes (Roche) by the Bio-Rad Blotting System. Next, the membranes were washed with Tris-buffered saline/Tween 20 (TBST) and blocked with 5% nonfat dry milk dissolved in TBST at room temperature for one hour. After three washes with TBST, the membranes were incubated with the primary antibodies (Supplementary Table 1) overnight at 4° C. After washing three times with TBST for 8 min each, the membranes were incubated for two hours at room temperature with the HRP-conjugated secondary antibody (Cell Signaling Technology). After three washes with TBST, the protein expression levels were evaluated with Western Lightning™ Chemiluminescence Reagent Plus (Life Technologies).

### *In vitro* functional studies

To evaluate the cell growth rate, tumor cells were seeded into 96-well plates (1,000 cells per well). After cell adherence, the changes in cell number were detected with a CCK-8 assay kit (Dojindo Corp. Japan) every day for 6 days according to the kit instructions. In the anchorage-dependent foci formation assay, tumor cells were seeded into 6-well plates (1,000 cells per well). The culture medium was changed every two days for two weeks. The cell foci were fixed with 75% ethyl alcohol and then stained with crystal violet staining solution (1%, Sigma). The number of foci were counted with AlphaEase FluorChem SP software after imaging. Anchorage-independent sphere formation in soft agar was used to assess the colony formation ability of the tumor cells. First, the bottoms of 6-well plates were overlaid with soft agar (0.6%). After solidification (approximately 30 min), the bottom layer of soft agar was subsequently covered with a tumor cell suspension in a soft agar mixture (5,000 cells per well in DMEM, 10% FBS and 0.4% soft agar). Next, the cells were cultured in a cell incubator for two weeks, and detectable spheres were imaged by microscope and counted in 10 views per well.

### Stem cell sphere formation assay

Briefly, 5 × 10^4^ cells were suspended in 500 μl of 1× DMEM/F12 medium (#12634010, Gibco) supplemented with 20 ng/ml EGF (#AF-100-15, PeproTech.), 10 ng/ml bFGF (#100-18B, PeproTech.), 4 μg/ml Insulin (#41-975-100, BIOIND), 1× B27 (#17504-044, Gibco), 1× Penicillin-Streptomycin Solution (#10378016, Gibco) and 0.5% Methyl Cellulose (#M0512, Sigma) and seeded in each well of 24-well ultralow-adsorption cell culture plates (#3473, Corning). Then, the cells were cultured at 37° C with 5% CO_2_. Every well was supplemented with 30 μl of normal medium every two days. Tumor spheres were completely formed in each well two weeks after seeding and were counted under an optical microscope with a 10× objective (total magnification 100×).

### *In vivo* transplantation tumor assay

The Animal Ethics Committee at Sun Yat-sen University Cancer Center approved the animal experiments. BALB/c nude mice (five-week-old, male) were purchased from the Guangdong Medical Laboratory Animal Center (Foshan, China) and were housed at the Experimental Animal Center in Sun Yat-sen University Cancer (Guangzhou, China). Tumor cells were suspended in 100 μl of DMEM and were transplanted subcutaneously into nude mice with a sterile injector. The length (L) and width (W) of the xenograft tumors were measured with calipers every week for four weeks. The tumor volumes were calculated as volume (mm^3^) = L × W^2^ × 0.5.

### Glucose consumption and lactate secretion assays

Hep3B cells (5 × 10^4^ cells) transfected with scramble vector, shRNA or *SNRPB*-rescue plasmids were seeded in 6-cm dishes and cultured with normal medium (DMEM + 10% FBS). After 48 hours, the cell culture supernatants were collected and centrifuged (3,000 rpm, 10 min) for metabolic analysis. The concentrations of glucose and lactic acid in the culture supernatants and fresh normal medium were analyzed with a Glucose Uptake Assay Kit (#ab136955, Abcam) and an L-Lactate Assay Kit (#ab65331, Abcam), respectively. Moreover, the total proteins were extracted from the cells with 1× RIPA Lysis Buffer (#ab156034, Abcam), and the protein concentrations were determined with a Pierce™ BCA Protein Assay Kit (#23227, Abcam) for relative quantification. Statistics: Glucose consumption = normal medium - culture supernatant; lactic acid secretion = culture supernatant - normal medium. Finally, the relative levels of glucose consumption and lactate secretion were divided by the corresponding total cell protein to eliminate the influence of the number of cells.

### Bioinformatics analyses

In this study, R software with the edgeR package was used to analyze the raw gene expression data from TCGA database (http://cancergenome.nih.gov/) [42]. The gene expression levels and survival data of HCC patients were obtained from the GEPIA website (http://gepia.cancer-pku.cn/) [43]. Two RNA sequencing datasets of Chinese HCC samples (GSE87630 and GSE36376) from the Gene Expression Omnibus (GEO) (https://www.ncbi.nlm.nih.gov/geo/) website were analyzed with the GEO2R module of the website [44]. The UALCAN (http://ualcan.path.uab.edu/) website was used to analyze the correlations between SNRPB expression and HCC grade or clinical stage [45]. To understand the cell functions and pathways affected by SNRPB expression in HCC, we performed Gene Ontology (GO) pathway enrichment analysis through the Coexpedia (http://www.coexpedia.org/) website [26].

### Statistical analysis

SPSS 23.0 (Chicago, IL) and GraphPad Prism 5.0 (San Diego, CA) software programs were used to analyze the research data. The correlation between *SNRPB* levels and clinicopathological features was analyzed by the Pearson χ^2^ test. Two-tailed, independent Student’s *t* test was used to evaluate the continuous data for any two groups. Cell growth curves were compared using a general curve model. A Cox partial proportional hazard regression model was used to perform univariate and multivariate analyses. Kaplan-Meier analysis with log-rank test was performed to calculate the prognostic value for HCC patients. When the *P* value < 0.05, the difference between results was statistically significant.

## Supplementary Material

Supplementary Figures

Supplementary Table 1
