# Supplementary material for: SNRPB-mediated RNA splicing drives tumor cell proliferation and stemness in hepatocellular carcinoma
Source: Aging (Albany NY). 2020 Dec 3;13(1):537–54. doi: 10.18632/aging.202164 (PMC7834993; doi:10.18632/aging.202164)
Supplement: Supplementary Figures [file aging-13-202164-s001.pdf]

SUPPLEMENTARY FIGURES

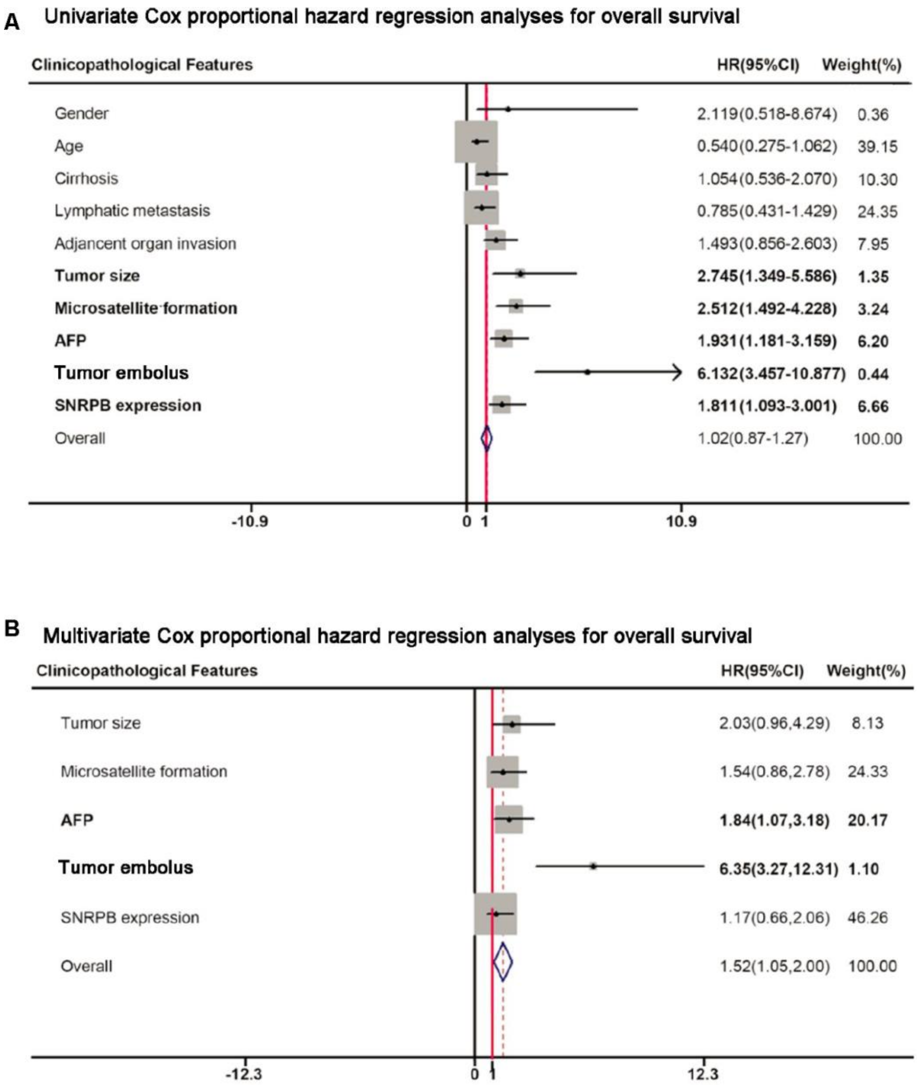

Supplementary Figure 1. SNRPB was associated with the poor survival of HCC patients. (**A**) Forrest plot of the univariate analysis shows that the hazard ratio (HR) of tumor size, microsatellite formation, AFP, tumor embolus, SNRPB expression are significantly greater than 1. (**B**) Forrest plot of the multivariate analysis shows that the hazard ratio (HR) of tumor embolus are significantly greater than 1.

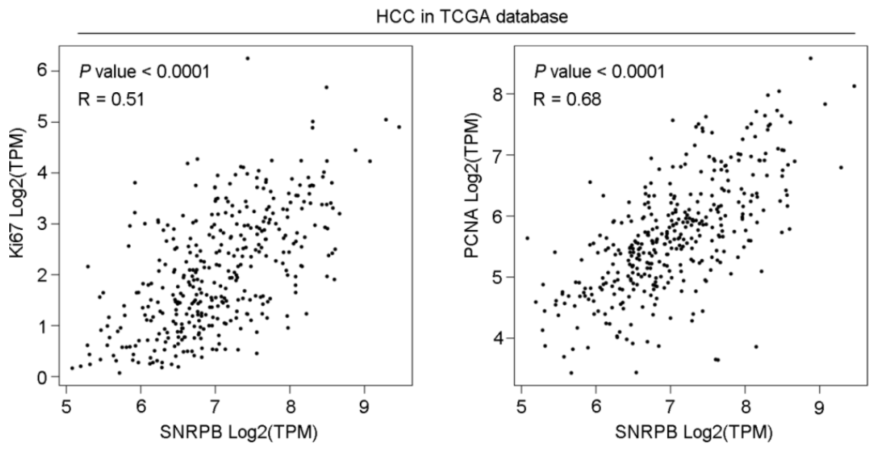

Supplementary Figure 2. The correlation of *SNRPB* expression and cell proliferation markers (*Ki67* and *PCNA*) in HCC based on TCGA database.

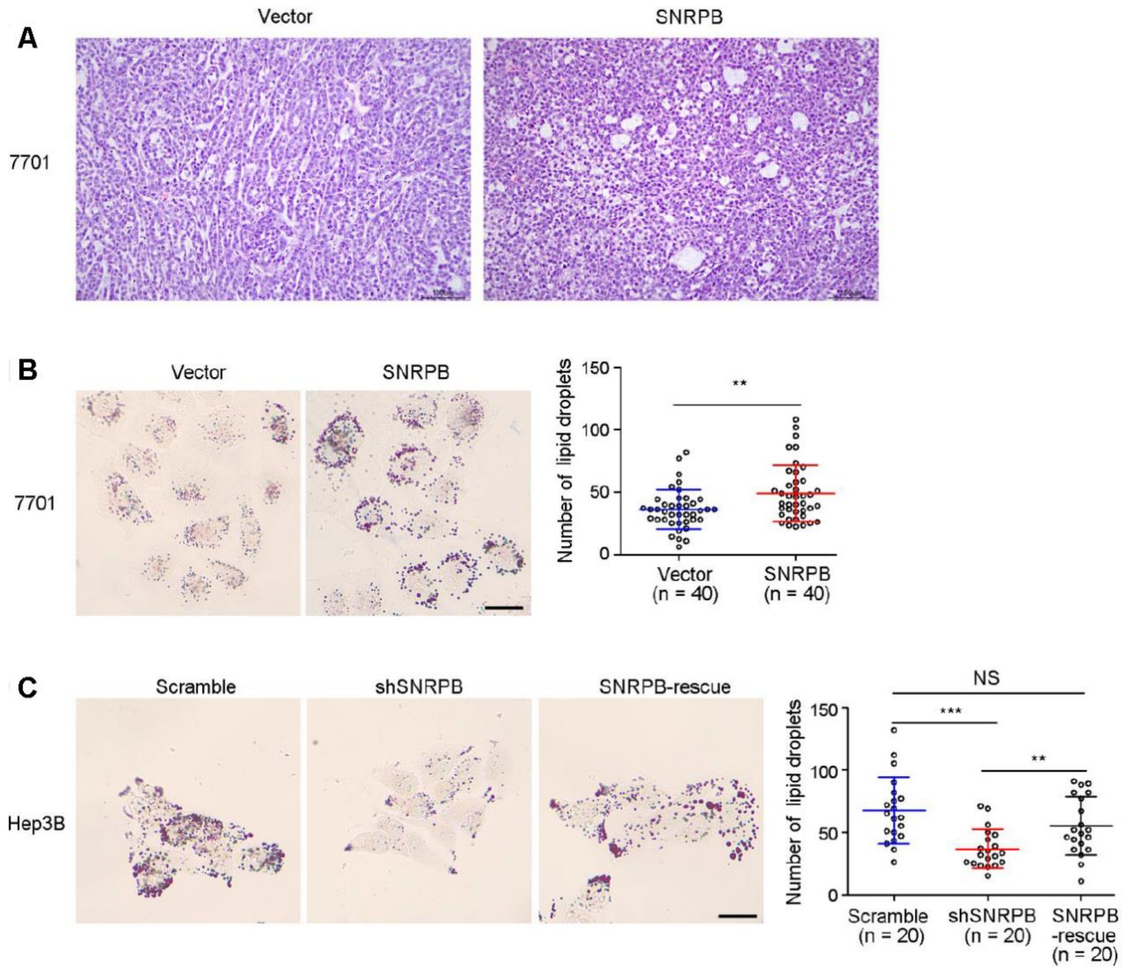

Supplementary Figure 3. SNRPB enhances the stemness of HCC cells. (**A**) Hematoxylin-eosin staining of xenografts from 7701 cells with or without *SNRPB* overexpression. Scale bar, 100 μm. (**B**, **C**) Oil Red O staining of 7701 cells after *SNRPB* overexpression and Hep3B cells when *SNRPB* expression was silenced or rescued. Scale bar, 50 μm. ***P* < 0.01, ****P* < 0.001, NS, no significant difference.

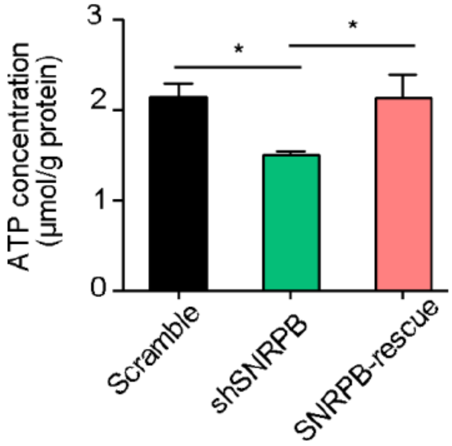

Supplementary Figure 4. The intracellular ATP concentration of Hep3B cells with *SNRPB* silence or rescue. *$P < 0.05$.
