# Supplementary material for: SNRPB-mediated RNA splicing drives tumor cell proliferation and stemness in hepatocellular carcinoma
Source: Aging (Albany NY). 2020 Dec 3;13(1):537–54. doi: 10.18632/aging.202164 (PMC7834993; doi:10.18632/aging.202164)
Supplement: Supplementary Table 1 [file aging-13-202164-s002.pdf]

SUPPLEMENTARY TABLE

Supplementary Table 1. Primary antibodies that used in western blot analysis.

ID	Antibodies	Corporations	Catalog	Dilutions
1	SNRPB	Santa Cruz Biotechnology	#sc-271094	1:1,000
2	CD133	Cell Signaling Technology	#64326	1:1,000
3	AFP	Cell Signaling Technology	#4448	1:1,000
4	CK19	Cell Signaling Technology	#4558	1:1,000
5	Nanog	Cell Signaling Technology	#4903	1:2,000
6	Sox2	Cell Signaling Technology	#3579	1:1,000
7	Phospho-Akt (Ser473)	Cell Signaling Technology	#4060	1:1,000
8	Akt (pan)	Cell Signaling Technology	#4685	1:1,000
9	Akt3	Cell Signaling Technology	#3788	1:1,000
10	β-Actin	Cell Signaling Technology	#3700	1:2,000
